# Point-of-care p24 antigen detection for early infant diagnosis of HIV infection: cross-sectional and longitudinal studies in Zambia

**DOI:** 10.1186/s12879-021-05808-2

**Published:** 2021-01-26

**Authors:** Catherine G. Sutcliffe, Jane Mutanga, Nkumbula Moyo, Abhishek K. Agarwal, Jessica L. Schue, Mutinta Hamahuwa, Kara M. Palamountain, Mark J. Fisher, Robert Elghanian, Philip E. Thuma, William J. Moss

**Affiliations:** 1 Johns Hopkins Bloomberg School of Public Health, 615 N. Wolfe St, Baltimore, MD 21205 USA; 2 Livingstone Central Hospital, Akapelwa St, Livingstone, Zambia; 3 Macha Research Trust, Choma, Zambia; 4 Center for Innovation in Global Health Technologies, Northwestern University, 2145 Sheridan Road, Evanston, IL 60208 USA; 5 Kellogg School of Management, Northwestern University, 2211 Campus Dr, Evanston, IL 60208 USA; 6 Present affiliation: RE Strong Scientific Consulting, 541 Lavergne Ave, Wilmette, IL 60091 USA

**Keywords:** Pediatric HIV, Diagnostics, Point-of-care, Sub-Saharan Africa

## Abstract

**Background:**

Early infant diagnosis of HIV infection is challenging in sub-Saharan Africa, particularly in rural areas, leading to delays in diagnosis and treatment. Use of a point-of-care test would overcome many challenges. This study evaluated the validity of a novel point-of-care p24 antigen detection test (LYNX) in rural and urban settings in southern Zambia.

**Methods:**

Two studies were conducted: a cross-sectional study from 2014 to 2015 at Macha Hospital (LYNX Hospital study) and a longitudinal study from 2016 to 2018 at 12 health facilities in Southern Province, Zambia (NSEBA study). In both studies, children attending the facilities for early infant diagnosis were enrolled and a blood sample was collected for routine testing at the central lab and immediate on-site testing with the LYNX test. The performance of the LYNX test was measured in comparison to nucleic acid-based testing at the central lab.

**Results:**

In the LYNX Hospital study, 210 tests were performed at a median age of 23.5 weeks (IQR: 8.9, 29.0). The sensitivity and specificity of the test were 70.0 and 100.0%, respectively. In the NSEBA study, 2608 tests were performed, including 1305 at birth and 1222 on children ≥4 weeks of age. For samples tested at birth, sensitivity was 13.6% (95% CI: 2.9, 34.9) and specificity was 99.6% (95% CI: 99.1, 99.9). While specificity was high for all ages, sensitivity increased with age and was higher for participants tested at ≥4 weeks of age (80.6%; 95% CI: 67.4, 93.7). Children with positive nucleic acid tests were more likely to be negative by the LYNX test if their mother received antiretroviral therapy during pregnancy (60.7% vs. 24.2%; *p* = 004).

**Conclusions:**

Considering the high specificity and moderate sensitivity that increased with age, the LYNX test could be of value for early infant diagnosis for infants ≥4 weeks of age, particularly in rural areas where centralized testing leads to long delays. Point-of-care tests with moderate sensitivity and high specificity that are affordable, easy-to-use, and easily implemented and maintained should be developed to expand access to testing and deliver same-day results to infants in areas where it is not feasible to implement nucleic acid-based point-of-care assays.

**Supplementary Information:**

The online version contains supplementary material available at 10.1186/s12879-021-05808-2.

## Background

In 2019, there were an estimated 1.8 million children living with HIV and 150,000 children were newly infected, with the majority living in sub-Saharan Africa [1]. Early diagnosis and treatment for these children is crucial to reduce the morbidity and mortality associated with HIV infection [2, 3]. However, early infant diagnosis is challenging, particularly in rural regions of sub-Saharan Africa, as virologic tests must be used and these tests are primarily available in central labs located in a few urban areas. The use of dried blood spots (DBS) has increased access to virologic testing but samples must be sent to the central labs and results returned to clinics. Turnaround times can be long outside of urban areas, leading to poor retention in care and delays in diagnosis and treatment [4–6]. Consequently, in 2019 only 69% of HIV-exposed infants in eastern and southern Africa and 33% of HIV-exposed infants in western and central Africa were estimated to have received a virologic test by 8 weeks of age [7]. As a result, only 58% of children living with HIV in eastern and southern Africa and 33% of children living with HIV in western and central Africa were receiving antiretroviral therapy (ART) [7].

A point-of-care test would overcome many of the current logistical and economic challenges to early infant diagnosis. Several tests have been developed [8], based on both nucleic acid and p24 antigen detection. Two tests, m-PIMA (Abbott Laboratories, Forest Park, IL; formerly Alere Q) and GeneXpert (Cepheid Inc., Sunnyvale, CA), both based on nucleic acid detection, are commercially available and on the World Health Organization’s list of prequalified diagnostic tests [9]. However, the technological and infrastructural requirements for these tests as well as their cost limit their utility. An affordable and simple test, one that could be used at smaller health facilities, would increase access to testing and treatment in more rural and remote areas of sub-Saharan Africa.

The LYNX test (Northwestern Global Health Foundation, Evanston, IL) is a low-cost and low-technology lateral flow assay based on p24 antigen detection that was designed for lower level and lower volume health facilities. In laboratory studies, the assay was found to have a sensitivity and specificity of 90–100% and 99–100%, respectively [10, 11]. In one clinical study among HIV-exposed infants 1–18 months of age at three peri-urban health centers in Mozambique, the assay was found to have a sensitivity and specificity of 71.9 and 99.6%, respectively [12]. No studies have evaluated the LYNX test at birth or in rural health centers where it was designed to be used.

The primary objective of this study was to evaluate the validity of the LYNX assay in comparison to standard-of-care nucleic acid-based testing among infants from birth to 18 months of age in health facilities in rural and urban Zambia. A secondary objective was to understand the feasibility of implementing a lateral flow assay in clinical and rural settings.

## Methods

### Overview and setting

The LYNX assay was evaluated in two studies conducted in Southern Province, Zambia: 1) an initial hospital-based study (LYNX Hospital Study) to evaluate the feasibility of implementing the LYNX assay in a rural setting; and 2) the Novel Screening for Exposed Babies (NSEBA) Study, a follow-up study to evaluate strategies for implementing point-of-care technologies for early infant diagnosis in Zambia, including a validation study of the LYNX assay at birth. In Southern Province, the HIV prevalence was estimated to be 13.3% among adults 15–59 years of age in 2016 [13]. During both studies, universal treatment of pregnant women living with HIV was implemented throughout the country, with infant prophylaxis evolving from nevirapine alone [14], to zidovudine plus nevirapine for 6 or 12 weeks, depending on the ART status of the mother in 2016 [15], and then to zidovudine plus lamivudine plus nevirapine for 6 or 12 weeks, depending on the ART status of the mother in 2018 [16]. For early infant diagnosis, HIV-exposed infants were recommended to be tested at 6 weeks and 6 months of age with a nucleic acid test, and at 12 months, 18–24 months of age and at least 6 weeks after breastfeeding cessation with a serologic test [15]. Testing at birth was introduced into the national guidelines in 2016 [15]. Nucleic acid-based testing was performed in designated central labs. Blood samples were collected on DBS cards and transported to the labs through transport systems established by the Ministry of Health. In the study area, the designated central lab was at Livingstone Central Hospital (estimated distance of 260 km away) and prior to the studies, the median turnaround time from sample collection to the result returning to the clinic was 54 days (range: 11, 176) and from sample collection to the result being returned to the mother was 92 days (range: 28, 487) [4].

### The LYNX assay

The LYNX assay was developed for early infant diagnosis by the Center for Innovation in Global Health Technologies at Northwestern University and the Northwestern Global Health Foundation. The assay is a low-cost (< 15 USD) dipstick p24 antigen lateral flow assay with a visual readout and requires 20–40 μL of plasma. A heat dissociation step separates bound antibodies from the p24 antigen. A combination of engineering and chemistry innovations overcomes sample gelling, which is often associated with sub-Saharan African infants due to inherently high concentration of antibodies in their plasma. The assay was designed to be performed in primary care clinics by healthcare workers in settings where power and refrigeration may not be available, and ambient temperatures may reach 35-40 °C. The LYNX assay is run with a battery-powered low-cost (2000 USD) compact instrument that is light-weight (1.52 kg) and portable, with the capacity to run one test at a time. The test is performed in eight steps, including the heat dissociation step, with results available in less than 60 min (Additional File 1).

### The LYNX hospital study: overview and procedures

The LYNX Hospital study was conducted between July 2014 and July 2015 at Macha Hospital, located in a rural area of Southern Province. Two LYNX instruments were placed at Macha Hospital and the five counselors involved in DBS collection at the HIV clinic and primary health center were trained to run the LYNX test. All of the counselors had a high school education and additional training in either HIV/ART counseling or phlebotomy. The counselors had a three-hour training session in the classroom by a trainer from the Northwestern Global Health Foundation and then conducted the LYNX test on study participants under observation by the trainer for 2 days. The two instruments were placed in the HIV clinic but were shared with and transported to the primary health center when an infant evaluated there required testing.

All HIV-exposed infants attending either the HIV clinic or primary health center at Macha Hospital for early infant diagnosis were eligible for enrollment. After enrollment, a questionnaire was administered to collect information on demographics, antenatal care, and prior HIV testing. Information on use of antiretroviral drugs by the mother or infant was abstracted from the medical record. As part of routine clinical care, a DBS card was collected. At the same time, a study blood sample (80 μL) was collected from participants and tested immediately with the LYNX test. The DBS was sent to the central lab at Livingstone Central Hospital for testing (Roche Amplicor HIV-1 DNA test v1.5 [Roche Diagnostics, Risch-Rotkreuz, Switzerland]). When the result from the central lab was returned to the clinic, the result and dates for each step in the process were documented by study staff.

### NSEBA study: overview and procedures

The NSEBA study was conducted at urban (UHC) and rural (RHC) health centers and hospitals in three areas in Southern Province (Table 1), including the Macha area (1 hospital, 4 RHCs), Choma Town (1 hospital, 1 UHC), and Livingstone City (1 hospital, 4 UHCs). The NSEBA study has been previously described [17]. Briefly, the study included two components: 1) an evaluation of testing at birth, which was a cross-sectional study at birth conducted at the study sites in Livingstone and Choma from June 2016 to April 2018; and 2) an evaluation of birth and routine testing within a longitudinal study conducted at the study sites in the Macha area from February 2016 to September 2018. While the national guidelines recommended testing at birth, this had only been implemented routinely at the hospital in Livingstone when the study began. Thus, at all other study sites, testing at birth was performed as part of the study.

**Table 1:** Description of NSEBA study sites in Southern Province, Zambia, 2016–2018

Study site	Area	Type of area	Study period (mm/yy)	Median (range) deliveries per week by women living with HIV	Median (range) DBS collected per week for EID	Eligible participants	Timing of follow-up	Staff trained to perform LYNX assay
Livingstone Central Hospital	Livingstone City	Urban	06/16–05/17 06/17–04/18	8 (1, 15)	N/A	All HEI High-risk HEI^a^	Birth only	1 Research staff^c^
Maramba UHC	Livingstone City	Urban	06/16–05/17 06/17–04/18	3 (0, 12)	N/A	All HEI High-risk HEI^a^	Birth only	1 Research staff^c^
Mahatma Gandhi UHC	Livingstone City	Urban	04/17–04/18	3 (0, 9)	N/A	High-risk HEI^a^	Birth only	1 Research staff^c^
Libuyu UHC	Livingstone City	Urban	04/17–04/18	1 (0, 5)	N/A	High-risk HEI^a^	Birth only	1 Research staff^c^
Choma General Hospital	Choma City	Urban	08/17–04/18	5 (2, 9)	N/A	High-risk HEI^a^	Birth only	2 Nurses
Shampande UHC	Choma City	Urban	08/17–04/18	2 (0, 5)	N/A	High-risk HEI^a^	Birth only	2 Nurses
Macha Hospital	Macha area	Rural	04/16–09/18	2 (0, 9)	3 (0, 11)	All HEI	Testing period^b^	5 Counselors 6 Research staff^c^
Mapanza RHC	Macha area	Rural	04/16–09/18	0 (0, 4)	1 (0, 7)	All HEI	Testing period^b^	1 Clinical Officer 2 Nurses 1 Counselor
Moboola RHC	Macha area	Rural	04/16–09/18	0 (0, 3)	1 (0, 7)	All HEI	Testing period^b^	2 Nurses 1 Counselor
Mangunza RHC	Macha area	Rural	04/16–09/18	0 (0, 3)	1 (0, 5)	All HEI	Testing period^b^	1 Nurse 1 Counselor
Nalube RHC	Macha area	Rural	04/16–07/17	0 (0, 2)	0 (0, 3)	All HEI	Testing period^b^	1 Nurse 1 Counselor

*DBS* Dried blood spots, *EID* Early infant diagnosis, *HEI* HIV-exposed infants, *N/A* Not applicable, *RHC* Rural health clinic, *UHC* Urban health center

^a^ High-risk defined as mothers receiving no antiretroviral drugs throughout pregnancy or starting to receive antiretroviral drugs during pregnancy to prevent mother-to-child transmission

^b^ Testing period includes any point of contact for early infant diagnosis testing from birth to post-weaning (e.g. birth, 6 weeks, 6 months, 12 months, 18 months, 24 months, post-weaning)

^c^ Research staff in Livingstone included 3 nurses; research staff in Macha included 1 nurse, 1 laboratory scientist, and 4 counselors by training

**Fig. 1:**
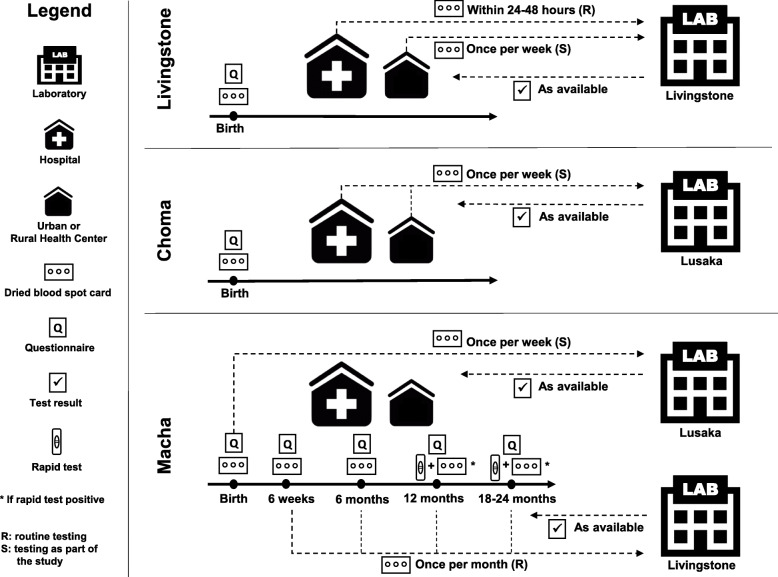
Overview of NSEBA study procedures by site

To evaluate testing at birth at study sites in Livingstone, Choma, and Macha, HIV-exposed infants who were delivered or brought for evaluation in the maternity wards were eligible for participation. In the first year of the study, all HIV-exposed infants were eligible for enrollment. In the second year of the study, enrollment in Livingstone and Choma was limited to infants at high risk of acquiring HIV (Table 1). Infants were eligible for enrollment up to the time of discharge from the site. HIV-exposed infants were identified by study staff through daily surveillance of admission and delivery registers and, if eligible, their mothers were approached for participation in the study. If willing to participate, women were asked to provide written informed consent and then administered a questionnaire to collect information on demographics, antenatal care, HIV testing history, and receipt of antiretroviral drugs. A DBS card was collected from the infant and, at the same time, a second whole blood sample was collected for immediate testing with the LYNX assay. The DBS card was sent to the central lab for testing (Fig. 1). Samples from the Livingstone sites were sent for testing in the central lab at Livingstone Central Hospital (COBAS® AmpliPrep/COBAS® TaqMan® Systems [Roche Diagnostics, Risch-Rotkreuz, Switzerland]) by study staff. Samples from the Choma and Macha sites, which were tested as part of the study, were sent by courier for testing in the Center for Infectious Disease Research in Zambia (CIDRZ; COBAS® AmpliPrep/COBAS® TaqMan® Systems [Roche Diagnostics, Risch-Rotkreuz, Switzerland]) in Lusaka. When results were received from the central lab, study staff contacted the participants by phone to alert them and request that they return to receive them.

To evaluate testing at all other time points after birth at the Macha sites, all HIV-exposed infants attending the study sites for early infant diagnosis at routine ages were eligible for participation and were then followed through to their post-weaning visit. At each visit, the same study procedures described above were performed, with routine testing performed at Livingstone Central Hospital (Fig. 1).

Results from the central lab in Livingstone and at CIDRZ were documented as well as the dates for each step in the testing process. At the end of the study period, results that were not yet back from either lab were retrieved from the lab database.

Study staff received 1 day of training by a trainer from the Northwestern Global Health Foundation on how to perform the LYNX assay. Study staff included both full-time research personnel hired for the study (Livingstone and Macha sites) and part-time personnel who provided EID services at the study sites (Choma and Macha sites). The qualifications of the staff performing the assay varied at each study site depending on the providers available who performed EID services (Table 1). After training, study staff at the RHCs in Macha and UHCs in Choma received weekly supervisory visits, and study staff at the Livingstone sites received quarterly supervisory visits by a supervisor from the Macha Hospital site. Two LYNX machines were placed at Macha Hospital and Livingstone Central Hospital. One LYNX machine was placed at all other study sites. When performing the LYNX assay, each step was documented and tests were interpreted immediately by study staff. If the test yielded an invalid result and the participant was still at the site, a second blood sample was collected and a second test was run.

In addition to the LYNX machines, imagers were placed at the sites to photograph the test strips after each test to understand how study staff were interpreting the test strip results. Photographs of a subset of tests with available results by September 2018 were sent to the Northwestern Global Health Foundation for review. The subset included all tests with a positive LYNX test or positive result from the central lab and a 10% random sample of negative tests. Two experts (AKA and KMP) independently read the photographed test strips. Where their results differed, they arrived at a final result by discussion and additional review.

### Statistical analysis

For both studies, the characteristics of study participants and turnaround times were summarized using descriptive statistics. For turnaround times in the NSEBA study, results that were not returned and later retrieved by study staff from the central lab were excluded.

The performance of the LYNX test was assessed by calculating the sensitivity, specificity, positive predictive value, and negative predictive value in comparison to nucleic acid-based testing at the central lab. In the NSEBA study, the performance of the LYNX test was assessed by age group (birth = 0–6 days; postnatal = 7–28 days; 6 weeks = 4–13 weeks; 6 months = 3–7 months; or all routine = 6 weeks, 6 months or > 7 months), receipt of ART to prevent mother-to-child transmission by mothers or children (routine ages only), location (hospitals or rural health centers), and operator qualifications (counselors, nurses/clinical officers, or research staff). The limited number of positive tests at birth from sites outside of Livingstone limited the analysis by location and operator; therefore, only results from post-birth visits in the Macha area are reported for this comparison. For measures reported for combined age groups, the jackknife method was used to estimate standard errors and confidence intervals to account for multiple tests per child. Binomial confidence intervals were calculated for measures calculated separately by age.

In the NSEBA study, the results of the LYNX test as interpreted by study staff were compared to the results as interpreted by the two experts by calculating a kappa statistic.

### Ethics statement

The LYNX Hospital and NSEBA studies were approved by the Institutional Review Boards at Macha Research Trust, the Johns Hopkins Bloomberg School of Public Health, the Ministry of Health in Zambia (LYNX Hospital study), and the National Health Research Authority in Zambia (NSEBA study). All parents or guardians provided written informed consent for participation in the study. As the LYNX test was not approved for clinical use in Zambia, the results were not provided to caregivers or healthcare providers in either study.

## Results

### LYNX hospital study (2014–2015)

**Fig. 2:**
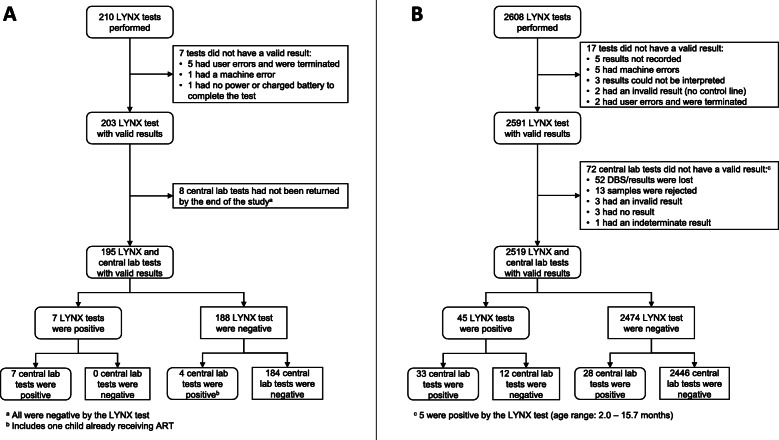
Study flowchart for **a** the LYNX Hospital study and **b** the NSEBA study

During the study period, 183 participants were enrolled and 210 LYNX tests were performed (Fig. 2a). The characteristics of study participants are presented in Table 2. For the 204 tests with a valid result (97.1%; see Fig. 2a for reasons a valid result was not obtained), the median time from blood collection to reading the test strip was 55 min (IQR: 54, 57). Valid results from the central lab were available for 195 tests (95.6%; Fig. 2a). Results were returned to the clinic a median of 75 days (IQR: 53, 104; range: 31, 320) after sample collection. Overall, 11 tests (5.6%) and 8 children (6.3%) were positive. One child, who tested positive by the central lab and negative by LYNX, was later found to be receiving ART and was excluded from further analysis. For the remaining 194 tests, the sensitivity and specificity of the LYNX test were 70.0% (95% confidence interval [CI]: 40.0, 99.9) and 100.0%, respectively. The positive and negative predictive values of the LYNX test were 100.0 and 98.3% (95% CI: 96.6, 100.0), respectively.

**Table 2:** Characteristics of study participants at enrollment and at each test

	LYNX Hospital Study		NSEBA Study	
	Participants (***n*** = 183)	Tests (***n*** = 210)	Participants (***n*** = 1857)	Tests (***n*** = 2608)
Sex – female, n (%)	97 (53.0)	107 (51.0)	936 (50.4)	1361 (52.2)
Age group^a^, n (%)				
Birth	0	0	1305 (70.3)	1305 (50.0)
Postnatal	3 (1.6)	3 (1.4)	81 (4.4)	81 (3.1)
6 weeks	74 (40.4)	74 (35.2)	247 (13.3)	502 (19.3)
6 months	84 (45.9)	107 (51.0)	167 (9.0)	607 (23.3)
> 7 months	22 (12.0)	26 (12.4)	57 (3.1)	113 (4.3)
All routine after birth	180 (98.4)	207 (98.6)	471 (25.4)	1222 (46.9)
Median (IQR; range) age				
Overall (in weeks)	17.3 (7.9, 28.9; 2.0, 60.6)	23.5 (8.9, 29.0)	0.1 (0.0, 4.6; 0.0, 68.6)	0.9 (0.1, 18.3; 0.0, 104.1)
Birth (in days)	–	–	0.4 (0.2, 0.8; 0.0, 6.7)	0.4 (0.2, 0.8; 0, 6.7)
Postnatal (in days)	15.0 (14.0, 19.0; 14.0, 19.0)	15.0 (14.0, 19.0; 14.0, 19.0)	10.0 (8.0, 14.0; 7.0, 26.0)	10.0 (8.0, 14.0; 7.0, 26.0)
6 weeks (in weeks)	7.3 (6.3, 10.1; 4.9, 12.9)	7.3 (6.3, 10.1; 4.9, 12.9)	7.1 (6.3, 8.4; 4.0, 12.9)	6.9 (6.3, 8.3; 4.0, 12.9)
6 months (in months)	6.1 (4.6, 6.7; 3.1, 7.9)	6.1 (5.3, 6.7; 3.1, 7.9	5.7 (4.0, 6.6; 3.0, 7.9)	6.1 (5.7, 6.7; 3.0, 7.9)
> 7 months (in months)	9.6 (8.3, 10.7; 8.1, 13.9)	9.4 (8.3, 10.7; 8.1, 13.9)	9.5 (8.5, 10.6; 8.0, 15.7)	9.4 (8.5, 10.7; 8.0, 23.9)
All routine after birth (in months)	4.1 (1.9, 6.6; 1.1, 13.9)	5.4 (2.2, 6.8; 1.1, 13.9)	2.7 (1.6, 6.2; 0.9, 15.7)	5.2 (1.7, 6.5; 0.9, 23.9)
Mother received ART during pregnancy, n (%)	162 (88.5)	185 (88.1)	1661 (89.5)	2367 (90.9)
Child received prophylactic ART after birth, n (%)^b^	153 (83.6)	178 (84.5)	791 (88.4)	1477 (89.8)
Mother currently receiving combination ART, n (%)^b^	174 (95.6)	201 (96.2)	840 (94.2)	1575 (95.9)

*ART* Antiretroviral therapy, *IQR* Interquartile range

^a^ Birth defined as 0–6 days; postnatal as 7–28 days; 6 weeks as 4–13 weeks; 6 months as 3–7 months; all routine after birth as 6 weeks, 6 months or > 7 months

^b^ Among participants tested after birth

### NSEBA study (2016–2018)

During the study period, 1857 participants were enrolled and 2608 LYNX tests were performed (Fig. 2b). The characteristics of study participants are presented in Table 2.

**Fig. 3:**
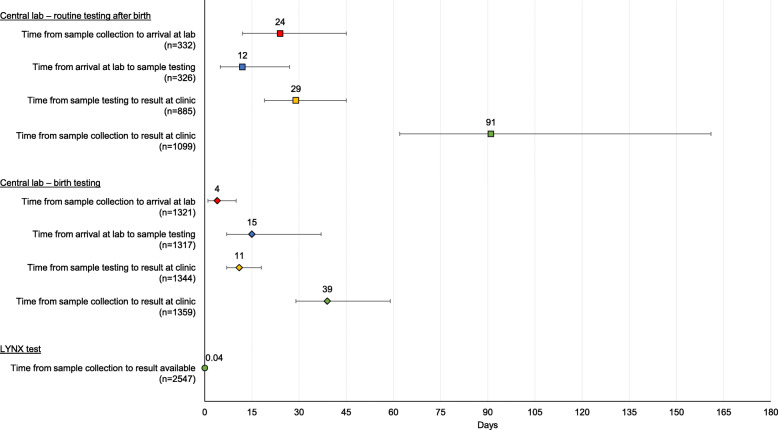
Turnaround times for the LYNX test and central labs. Note: Point estimates represent medians and error bars represent interquartile ranges

The median ambient temperature while running the LYNX tests was 26 °C (IQR: 24, 28; range: 14, 39). For 1946 (75.7%) tests, power was available at the site for the duration of testing. For 37 (1.4%) and 588 (22.9%) tests, power was available for some time or not at all while testing, respectively, with the test performed using batter power (power availability was missing for 37 tests). A valid result was obtained for 2591 (99.3%; see Fig. 2b for reasons a valid result was not obtained) LYNX tests (Fig. 2b). No protocol deviations were noted for the invalid tests and the result was interpreted from the test strip. The median time from sample collection (*n* = 2547) to a valid result was 54 min (IQR: 53, 56) (Fig. 3), with a maximum time of 147 min for a participant who had an initial invalid result.

Samples were sent to the central lab for all 2591 samples. Valid results were available for 2519 (97.2%) samples and 1834 (98.2%) children (Fig. 2b). For 51 (2.0%) samples, results were not received at the site and were retrieved from the lab database. For samples collected from the maternity wards, the median turnaround from sample collection to the result arriving at the clinic was 39 days (IQR: 29, 59; range: 6, 403) (Fig. 3). A total of 917 caregivers (67.3%) were given their results a median of 60 days (IQR: 44, 91; range: 11, 730) after sample collection. For routinely collected samples after birth, the median turnaround time from sample collection to the result arriving at the clinic (either by text message or hardcopy) was 91 days (IQR: 62, 161; range: 7, 612) (Fig. 3). A total of 954 (82.5%) caregivers were given their results a median of 161 days (IQR: 93, 217; range: 25, 687) after sample collection. Overall, 61 tests (2.4%) and 54 children (2.9%) were positive during the study period, including 22 (1.7%) tests/children at birth and 36 (3.1%) tests and 31 (4.1%) children at ≥4 weeks of age.

**Fig. 4:**
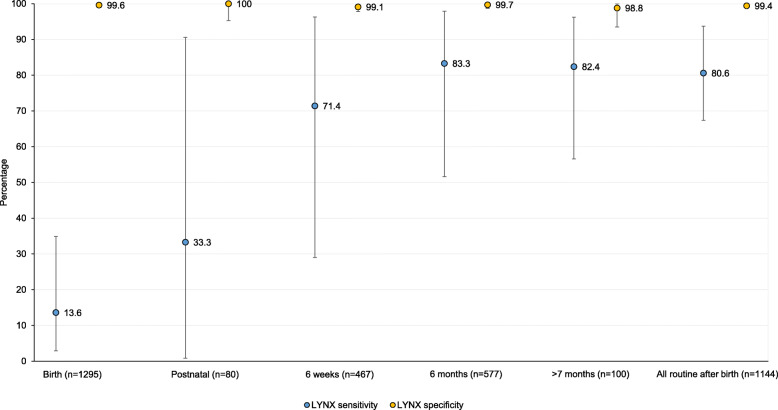
Sensitivity and specificity of the LYNX test by age^**a**^**.** ^a^ Birth = 0–6 days; postnatal = 7–28 days; 6 weeks = 4–13 weeks; 6 months = 3–7 months; all routine after birth = 6 weeks, 6 months or > 7 months. Note: Error bars represent 95% confidence intervals. One child who initially tested positive by the central lab and negative by the LYNX test at 7.9 weeks of age was found to be HIV uninfected after confirmatory testing at multiple visits, increasing sensitivity of the LYNX tests at 6 weeks to 83.3 and 82.9% at all routine visits after birth

The performance of the LYNX test varied by the child’s age at testing (Fig. 4; Additional File 2). For samples tested at birth, the sensitivity was 13.6% (95% CI: 2.9, 34.9) and the specificity was 99.6% (95% CI: 99.1, 99.9). While specificity was high for all ages, the sensitivity increased with age and was higher for participants tested at a routine visit after birth (≥4 weeks of age). At routine ages after birth, the sensitivity was 80.6% (95% CI: 67.4, 93.7) and the specificity was 99.4% (95% CI, 98.9, 99.9). One child who tested positive by the central lab and negative by LYNX at 7.9 weeks of age was negative by both tests on confirmation and deemed HIV uninfected. Taking this result into account increased the sensitivity of LYNX at routine ages after birth to 82.9% (95% CI, 70.2, 95.5). Results at routine ages after birth did not vary significantly by location or operator (Additional File 2).

Among the 61 tests positive at the central lab, those with discordant results by LYNX were significantly more likely to come from a child whose mother received ART during pregnancy (60.7% vs. 24.2%; *p* = 0.04). Consequently, the sensitivity of the LYNX test was significantly higher (69.4% vs. 32.0%) for tests conducted when the mother had not received ART to prevent mother-to-child transmission (Additional File 2). Those with discordant results by LYNX at routine ages after birth were also more likely to come from a child who received prophylactic ART (62.5% vs. 38.7%; *p* = 0.23), although this difference was not statistically significant.

A subset of 246 tests were sent for expert review, including 41 tests positive by LYNX and 41 positive at the central lab. There was good agreement between the study staff and experts (kappa = 0.65; Additional File 3A), although the experts were more likely to determine a test to be either invalid (7.8% of results considered valid by study staff were recorded as invalid by the experts) or positive (4.4% of negative results by study staff were recorded as positive by the experts). Experts designated tests to be invalid if (a) contamination was present on the sample pad, (b) a discontinuous control and/or test line was present on the test strip, (c) the intensity of the test line was darker in comparison to the control line on the test strip, and (d) there was a gradient intensity across the width of the test strip, which differed between the control and test line. Among the 216 tests with valid results by all three methods (the central lab, LYNX review by study staff, and LYNX review by the experts), the agreement with the central lab was higher for the experts (kappa = 0.78; Additional File 3B) than study staff (kappa = 0.69; Additional File 3C).

## Discussion

The LYNX p24 antigen test was found to have moderate sensitivity and high specificity among infants 4 weeks of age and older. However, the sensitivity of the LYNX test at birth was low. The test was robust and worked well in these challenging settings, accommodating high ambient temperatures in the clinics and power outages. In addition, the test was simple enough to be performed by clinic staff of all levels, including laboratory staff, nurses, and counselors. This contrasts with current point-of-care nucleic acid-based tests that are unlikely to be performed in many rural health centers due to requirements for trained laboratory personnel and routine maintenance, and vulnerabilities to high ambient temperatures and power outages.

The sensitivity of the LYNX test at routine ages after birth was found to be 70–80% in the LYNX Hospital and NSEBA studies. This is similar to the sensitivity of 71.9% observed in a clinical study of the LYNX assay in Mozambique conducted in 2013–2014 among infants 1–18 months of age [18] but lower than the sensitivities reported from laboratory studies (90–100%) [10, 11]. Similar observations of lower performance in the clinic than the laboratory have been reported for the HIV rapid test [19, 20], which is a similar lateral flow assay, and may be due to differences in storage conditions and protocols used in the clinic, including storage in non-climate controlled rooms, deviations in the protocol when performing the assay, and errors in interpretation of the results of the assay. The sensitivity of the LYNX test was also lower than currently available nucleic acid-based point-of-care (or near point-of-care) tests (96–99% in clinical studies) [21]. Reasons for the lower sensitivity include the lower efficiency of signal amplification in immunoassays as compared to nucleic-acid tests and lower levels of p24 antigenemia in recently infected infants, which may lead to discrepancies between these two types of assays. HIV RNA levels are known to appear and increase more rapidly after transmission than p24 antigen, leading to a shorter window period to detection [22]. Given the kinetics of nucleic acid and p24 antigen, it is therefore not surprising that the LYNX test had a lower sensitivity when used at birth and in the postnatal period in this study, as a larger proportion of infants are newly or recently infected at this time point [23]. This finding is consistent with older studies that found lower sensitivity of p24 antigen assays compared to PCR at or shortly after birth [24, 25].

Given the visual format of the LYNX test, the result for each test was subject to interpretation by the user, which ultimately impacted the performance of the assay. Differences in interpretation may have occurred due to differences in the strength of the control and patient lines on the test strip, lighting in the room where the test strip was being read, eyesight of the user, and user training. The extent of variability in interpretation was assessed through a second, expert reader who was found to be more likely to accurately assess positive results and judge an assay to be invalid than study staff. The study from Mozambique that evaluated the LYNX test also found variability in interpretation based on the user, with three operators reporting a sensitivity of 0–60% and four operators reporting a sensitivity of 80–100% [18]. This contributed to moderate overall sensitivity of the assay (71.9%) [18]. These results emphasize the need for ongoing training and supervision after implementation of any point-of-care test to ensure that protocols are followed for performing the assay and reading the result. These findings can also inform optimization of current assays or development of new assays with a similar format, as they suggest that use of an automated reader could minimize or eliminate this potential source of error and increase consistency and sensitivity.

With a strong PMTCT program and adoption of Option B+, the rate of mother-to-child transmission has decreased in Zambia [7], such that the number of HIV-infected infants identified in this study was small. This led to wide confidence intervals around the estimates of sensitivity and specificity and limited evaluation of performance by location and operator. This will be a challenge for any clinical study evaluating the performance of new assays for EID in the future.

Given turnaround times of 1.5–3 months for the central lab to return results to the clinics and the substantial number of results that were not returned to mothers, particularly at birth, there is a need for point-of-care tests to facilitate EID and linkage to care in these settings. The LYNX test produced results in less than an hour and performed consistently in a range of clinic environments. Despite moderate sensitivity, the LYNX test could still play an important role in diagnosing infants after the postnatal period (≥4 weeks of age) for smaller and more remote clinics where current point-of-care nucleic acid tests cannot be performed. While the low sensitivity at birth would not make the LYNX test effective for diagnosing infants at that time point, in settings of high PMTCT coverage such as Zambia where only ~ 1% of infants will be positive at birth [17], it may be more cost-effective to initiate testing at 6 weeks of age, when the LYNX test demonstrated moderate sensitivity, and strengthen retention and linkage to care [26]. Nucleic acid-based assays have dominated the pipeline for point-of-care tests due to their analytic performance. However, current nucleic acid-based point-of-care machines are approximately 10 times more expensive than the LYNX machine and some have requirements for infrastructure and human resources that make it challenging to place them in smaller and more remote settings. Thus, there will still be a need with these assays to refer infants for testing from clinics that cannot accommodate the requirements of these machines or to create decentralized hub-and-spoke models for testing. Both options create the potential for poor retention of infants during the testing period and failure to link infected infants to care and treatment in a timely manner. A test with moderate sensitivity and high specificity that is affordable, easy-to-use, and easily implemented and maintained, like the LYNX test, could still be useful if it can expand access to testing and deliver same-day results to infants in areas where it is not feasible to implement nucleic acid-based point-of-care assays. On-site point-of-care testing can lead to > 98% of mothers receiving results and > 66% of HIV-infected infants initiating ART on the same day as sample collection [27, 28]. Consequently, tests may need to be evaluated not only on their performance but also on their ability to achieve their intended benefit. As shown in a modeling exercise, an assay with only 80% sensitivity that results in 99% of positive infants linked to care achieves the same level of ART coverage as an assay with 95% sensitivity that results in only 85% of positive infants linked to care [29].

## Conclusions

In summary, the LYNX test was found to have low sensitivity at birth but moderate sensitivity after 4 weeks of age. Given its high specificity, affordability, and robustness, the LYNX test could still play an important role in EID to increase linkage to care for HIV-infected infants ≥4 weeks of age at smaller clinics and in remote areas where implementing current nucleic acid-based tests is not feasible. In addition, the lessons learned about the challenges and utility of this type of point-of-care assay in this setting are valuable for informing development of new diagnostic assays for EID, HIV, and other infections such as SARS-CoV-2.

## Supplementary Information

**Additional file 1.** Procedures for performing the LYNX test. Note: Figure provided courtesy of the Northwestern University Center for Innovation in Global Health Technologies and the Northwestern Global Health Foundation.

**Additional file 2.** Performance characteristics of the LYNX test in comparison to nucleic acid-based testing at the central lab, overall and by age, PMTCT status, location and operator in the NSEBA study.

**Additional File 3.** A. Comparison of interpretation of LYNX tests between study staff and expert review in the NSEBA study. B. Comparison of test results between LYNX as read by study staff and the central lab in the NSEBA study. C. Comparison of test results between LYNX as read by experts and the central lab in the NSEBA study.

## Data Availability

Under the Research Health Act, the Government of Zambia does not allow public access to data collected in Zambia. All investigators interested in the data are required to submit a written request to the Ministry of Health. Contact Dr. Catherine Sutcliffe (csutcli1@jhu.edu) to coordinate the request.

## Abbreviations

ART: Antiretroviral therapy
CI: confidence interval
CIDRZ: Center for Infectious Disease Research in Zambia
DBS: Dried blood spots
EID: Early infant diagnosis
HEI: HIV-exposed infants
HIV: Human immunodeficiency virus
IQR: Interquartile range
N/A: Not applicable
NSEBA: Novel Screening for Exposed Babies Study
PMTCT: Prevention of mother to child transmission of HIV
UHC: Urban health center
RHC: Rural health center
USD: United States dollar
