# Supplementary material for: Point-of-care p24 antigen detection for early infant diagnosis of HIV infection: cross-sectional and longitudinal studies in Zambia
Source: BMC Infect Dis. 2021 Jan 26;21:118. doi: 10.1186/s12879-021-05808-2 (PMC7835654; doi:10.1186/s12879-021-05808-2)
Supplement: Supplementary file 1 — Additional file 1. Procedures for performing the LYNX test. Note: Figure provided courtesy of the Northwestern University Center for Innovation in Global Health Technologies and the Northwestern Global Health Foundation. [file 12879_2021_5808_MOESM1_ESM.docx]

**Additional File 1. Procedures for performing the LYNX test**


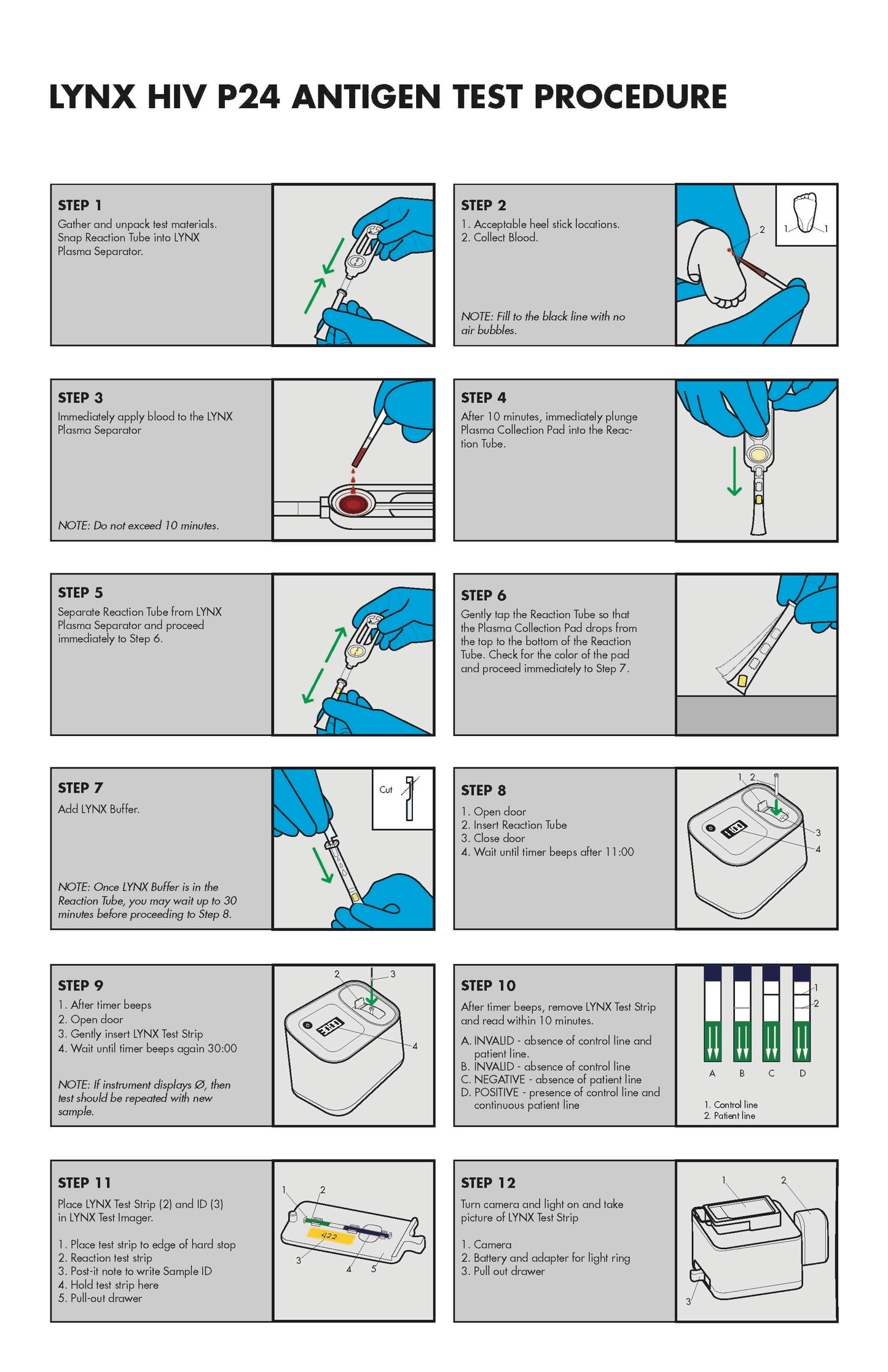


Note: Figure provided courtesy of the Northwestern University Center for Innovation in Global Health Technologies and the Northwestern Global Health Foundation
