# Supplementary material for: Point-of-care p24 antigen detection for early infant diagnosis of HIV infection: cross-sectional and longitudinal studies in Zambia
Source: BMC Infect Dis. 2021 Jan 26;21:118. doi: 10.1186/s12879-021-05808-2 (PMC7835654; doi:10.1186/s12879-021-05808-2)
Supplement: Supplementary file 2 — Additional file 2. Performance characteristics of the LYNX test in comparison to nucleic acid-based testing at the central lab, overall and by age, PMTCT status, location and operator in the NSEBA study. [file 12879_2021_5808_MOESM2_ESM.docx]

**Additional File 2. Performance characteristics of the LYNX test in comparison to nucleic acid-based testing at the central lab, overall and by age, PMTCT status, location, and operator in the NSEBA study**

| **Visit timing^a^** | **# tests** | **# positive**  **by central lab** | | **# negative**  **by central lab** | | **Sensitivity** | **Specificity** | **Positive predictive value** | **Negative predictive value** |
| --- | --- | --- | --- | --- | --- | --- | --- | --- | --- |
|  |  | **# positive by LYNX** | **# negative by LYNX** | **# positive by LYNX** | **# negative by LYNX** |  |  |  |  |
| **Overall** | | | | | | | | | |
| Birth | 1295 | 3 | 19 | 5 | 1268 | 13.6% | 99.6% | 37.5% | 98.5% |
| Postnatal | 80 | 1 | 2 | 0 | 77 | 33.3% | 100.0% | 100.0% | 97.5% |
| 6 weeks | 467 | 5 | 2^b^ | 4 | 456 | 71.4% | 99.1% | 55.6% | 99.6% |
| 6 months | 577 | 10 | 2 | 2 | 563 | 83.3% | 99.7% | 83.3% | 99.7% |
| >7 months | 100 | 14 | 3^c^ | 1 | 82 | 82.4% | 98.8% | 93.3% | 96.5% |
| All routine after birth | 1144 | 29 | 7^b^ | 7 | 1101 | 80.6% | 99.4% | 80.6% | 99.4% |
| All | 2518 | 33 | 28 | 12 | 2446 | 54.1% | 99.5% | 73.3% | 98.9% |
| **By receipt of PMTCT by the mother** | | | | | | | | | |
| No | 223 | 25 | 11 | 2 | 185 | 69.4% | 98.9% | 92.6% | 94.5% |
| Birth | 115 | 2 | 7 | 0 | 108 | 22.2% | 100% | 100.0% | 93.9% |
| All routine after birth | 92 | 22 | 3 | 2 | 65 | 88.0% | 97.0% | 91.7% | 95.6% |
| Yes | 2290 | 8 | 17 | 10 | 2255 | 32.0% | 99.6% | 44.4% | 99.3% |
| Birth | 1177 | 1 | 12 | 5 | 1159 | 7.7% | 99.6% | 16.7% | 99.0% |
| All routine after birth | 1047 | 7 | 4 | 5 | 1031 | 63.6% | 99.5% | 58.3% | 99.6% |
| **By receipt of PMTCT by the child among tests conducted at routine visits after birth** | | | | | | | | | |
| No | 137 | 18 | 3 | 2 | 114 | 85.7% | 98.3% | 90.0% | 97.4% |
| Yes | 996 | 11 | 4 | 5 | 976 | 73.3% | 99.5% | 68.8% | 99.6% |
| **By location and operator in the Macha area at routine visits after birth** | | | | | | | | | |
| Location - Hospital | 601 | 16 | 3^b^ | 6 | 576 | 84.2% | 99.0% | 72.7% | 99.5% |
| Location - RHCs | 539 | 13 | 4 | 1 | 521 | 76.5% | 99.8% | 92.9% | 99.2% |
| Operator - Counselors | 648 | 21 | 5^b^ | 4 | 618 | 80.8% | 99.4% | 84.0% | 99.2% |
| Operator - Nurses/clinical officers | 156 | 3 | 0 | 0 | 153 | 100.0% | 100.0% | 100.0% | 100.0% |
| Operator - Research staff | 308 | 5 | 1 | 3 | 299 | 83.3% | 99.0% | 62.5% | 99.7% |

PMTCT: prevention of mother-to-child transmission

^a^ Birth = 0-6 days; postnatal = 7-28 days; 6 weeks = 4-13 weeks; 6 months = 3-7 months; all routine after birth = 6 weeks, 6 months or >7 months.

^b^ One child was found to be HIV uninfected after confirmatory testing at multiple visits, increasing sensitivity at 6 weeks to 83.3% and 82.9% at all routine visits after birth.

^c^ One child was LYNX positive at a later visit.
