# Supplementary material for: Point-of-care p24 antigen detection for early infant diagnosis of HIV infection: cross-sectional and longitudinal studies in Zambia
Source: BMC Infect Dis. 2021 Jan 26;21:118. doi: 10.1186/s12879-021-05808-2 (PMC7835654; doi:10.1186/s12879-021-05808-2)
Supplement: Supplementary file 3 — Additional File 3. A. Comparison of interpretation of LYNX tests between study staff and expert review in the NSEBA study. B. Comparison of test results between LYNX as read by study staff and the central lab in the NSEBA study. C. Comparison of test results between LYNX as read by experts and the central lab in the NSEBA study. [file 12879_2021_5808_MOESM3_ESM.docx]

**Additional File 3.**

1. **Comparison of interpretation of LYNX tests between study staff and expert review in the NSEBA study**

| **LYNX result by expert review** | **LYNX result by study staff** | | | |
| --- | --- | --- | --- | --- |
|  | **Positive** | **Negative** | **Invalid** | **Total** |
| **Positive** | 34 (13.8) | 9 (3.7) | 1 (0.4) | 44 (17.9) |
| **Negative** | 1 (0.4) | 182 (74.0) | 0 | 183 (74.4) |
| **Invalid** | 6 (2.4) | 13 (5.3) | 0 | 19 (7.7) |
| **Total** | 41 (16.7) | 204 (82.9) | 1 (0.4) | 246 (100.0) |

Note: Kappa = 0.65 (95% confidence interval: 0.55, 0.76)

1. **Comparison of test results between LYNX as read by study staff and the central lab in the NSEBA study**

| **Central lab test result** | **LYNX result by study staff** | | |
| --- | --- | --- | --- |
|  | **Positive** | **Negative** | **Total** |
| **Positive** | 25 (11.6) | 13 (6.0) | 38 (17.6) |
| **Negative** | 5 (2.3) | 173 (80.1) | 178 (82.4) |
| **Total** | 30 (13.9) | 186 (86.1) | 216 (100.0) |

Note: Kappa = 0.69 (95% confidence interval: 0.55, 0.82)

1. **Comparison of test results between LYNX as read by experts and the central lab in the NSEBA study**

| **Central lab test result** | **LYNX result by expert review** | | |
| --- | --- | --- | --- |
|  | **Positive** | **Negative** | **Total** |
| **Positive** | 31 (14.4) | 7 (3.2) | 38 (17.6) |
| **Negative** | 7 (3.2) | 171 (79.2) | 178 (82.4) |
| **Total** | 38 (17.6) | 178 (82.4) | 216 (100.0) |

Note: Kappa = 0.78 (95% confidence interval: 0.66, 0.89)
